# Increased thickness and decreased blood flow velocity of the choroid in a patient with acute macular neuroretinopathy

**DOI:** 10.1186/s12886-019-1123-0

**Published:** 2019-05-14

**Authors:** Yuki Hashimoto, Wataru Saito, Michiyuki Saito, Yuka Hasegawa, Susumu Ishida

**Affiliations:** 1 Department of Ophthalmology, Faculty of Medicine and Graduate School of Medicine, Hokkaido University, Nishi 7, Kita 15, Kita-ku, Sapporo, 060-8638 Japan; 2 Kaimeido Eye and Dental Clinic, Sapporo, Japan

**Keywords:** Acute macular neuroretinopathy, Choroidal blood flow velocity, Choroidal thickness, Enhanced depth imaging optical coherence tomography, Laser speckle flowgraphy, Mean blur rate

## Abstract

**Background:**

The involvement of choroidal lesions in acute macular neuroretinopathy (AMN) is not yet fully understood. We quantitatively examined sequential changes in the morphology and circulation hemodynamics of the choroid using enhanced depth imaging optical coherence tomography (EDI-OCT) and laser speckle flowgraphy (LSFG) in a patient with AMN.

**Case presentation:**

A 15-year-old boy was referred to our hospital due to AMN in his right eye alone. The next day AMN developed in his left eye. Three months later, AMN lesions in both eyes spontaneously resolved and the morphology of macular photoreceptors improved. Using EDI-OCT, central choroidal thickness (CCT) was examined for a period of three months, starting from the initial visit. Using LSFG, macular mean blur rate (MBR) was examined for three months, starting 1 week after the initial visit. At the first visit, CCT of the right eye with AMN was 82 μm higher than that of the left eye, which had not yet developed AMN, and decreased by 86 μm after three months. In the left eye, similarly, CCT increased by 16 μm after the AMN onset at 1 week compared with a pre-onset value at the first visit and thereafter decreased by 57 μm at 3 months. Macular MBR increased by 20–55% OD and 51–71% OS during the follow-up until 3 months.

**Conclusions:**

We found that the choroid at the macula thickened at the onset of AMN and became thin with the regression of disease. Therefore, in concert with MBR data, these results further strengthened our hypothesis that choroidal circulation impairment plays a role in the pathogenesis of AMN.

## Background

Acute macular neuroretinopathy (AMN) is a rare disease characterized by a wedge-shaped dark reddish-brown lesion at the macula [1]. Prevalence and etiology of AMN are not fully clarified, although approximately 100 cases have been previously reviewed [2]. In patients with AMN, spectral domain optical coherence tomography (SD-OCT) revealed that the visual dysfunctions in AMN were possibly caused by the impairment of photoreceptors [3, 4]. A recent study using OCT angiography (OCTA) demonstrated a flow void of the choriocapillaris corresponding to the AMN lesion, suggesting ischemia of the choriocapillaris [5]. OCTA and SD-OCT both appear to be useful in differentiating AMN from paracentral acute middle maculopathy; however, the mechanism underlying photoreceptor impairment in AMN is still unclear.

Choroidal thickness and choroidal blood flow velocity are parameters that are capable of evaluating activity of various chorioretinal diseases. In healthy subjects, both thickness and blood flow velocity in the choroid decreased due to decrease of ocular perfusion pressure (OPP) during elevation of intraocular pressure, suggesting that there is a reliable relationship between the parameters [6]. However, we have shown that the parameters have distinctive patterns depending on disease types. In choroiditis such as in Vogt-Koyanagi-Harada disease and serpiginous choroiditis, choroidal thickness decreased and choroidal blood flow velocity increased with regression of diseases (inflammatory pattern) [7, 8]. A series of other diseases in the acute zonal occult outer retinopathy complex, unilateral acute idiopathic maculopathy, and acute posterior multifocal placoid pigment epitheliopathy also showed an inflammatory pattern [9–17]. In contrast, the thickness and blood flow velocity concurrently decreased with regression of acute central serous chorioretinopathy (sympathetic pattern) [18, 19]. Moreover, both of the parameters increased with regression of commotio retinae (vaso-occlusive pattern) [20, 21]. Importantly, changes in the thickness and blood flow during disease regression correlated inversely in Vogt-Koyanagi-Harada disease and positively in central serous chorioretinopathy [7, 19]. Thus, investigation into thickness-flow patterns in the choroid may contribute to elucidation of disease pathogenesis.

Laser speckle flowgraphy (LSFG) demonstrated that choroidal blood flow velocity decreased at the lesion sites in patients with AMN during the acute stage and increased with regression of this disease [14, 15]. These results suggest that choroidal circulation impairment is involved in the pathogenesis of this disease. In an eye with AMN [15], choroidal thickness at the AMN lesion site was greater than the value in the unaffected fellow eye; however, it has not been determined whether the choroid thickened in the acute stage of this disease or originally had greater thickness than the fellow eye prior to the onset of AMN. To further investigate the relationship between the choroid and photoreceptor impairments in AMN, we examined changes in morphology and circulation hemodynamics of the choroid using enhanced depth imaging OCT (EDI-OCT) and LSFG in a patient with AMN whose choroidal thickness could be evaluated from one day before the onset of AMN.

## Case presentation

A 15-year-old boy complained of central vision loss in his right eye, which persisted for 2 weeks. The patient’s medical and family histories were unremarkable.

**Fig. 1:**
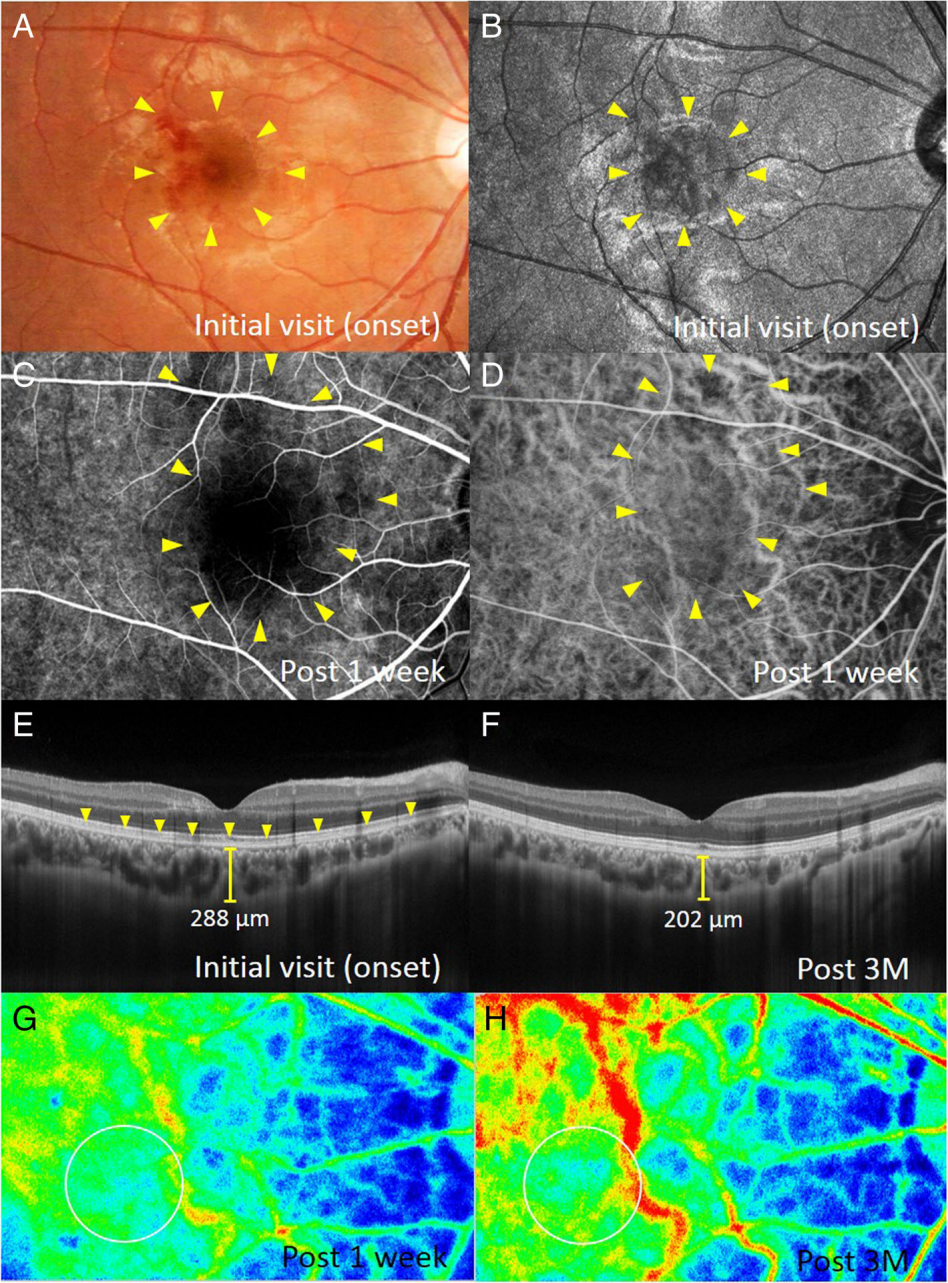
Images of the right eye at the first visit (**a**, **b**, **e**), 1 week (**c**, **d**, **g**), and 3 months after (**f**, **h**) the first visit in a patient with bilateral acute macular neuroretinopathy (AMN). **a** Fundus photograph shows a wedge-shaped dark reddish lesion (arrows) in the macula. **b**-**d** Corresponding to the macular lesion, scanning laser ophthalmoscope (SLO) infrared imaging shows the dark area (**b**, arrows) and initial-phase fluorescein angiography (**c**) and late-phase indocyanine green angiography (**d**) shows hypofluorescence (arrows). **e** A horizontal enhanced depth imaging optical coherence tomography (EDI-OCT) image through the fovea shows the loss or disruption of the interdigitation zone (IZ) at the macula (arrows). Central choroidal thickness (CCT) is 288 μm. **f** Macular IZ was restored, and CCT decreased to 202 μm. **g**, **h** Composite color map images of the mean blur rate (MBR) measured by laser speckle flowgraphy (LSFG). The blue color indicates low MBR, while the red color does high MBR. MBR within the circle at 3 months (**h**) increased to 40.0% compared with that at 1 week (**g**)

**Fig. 2:**
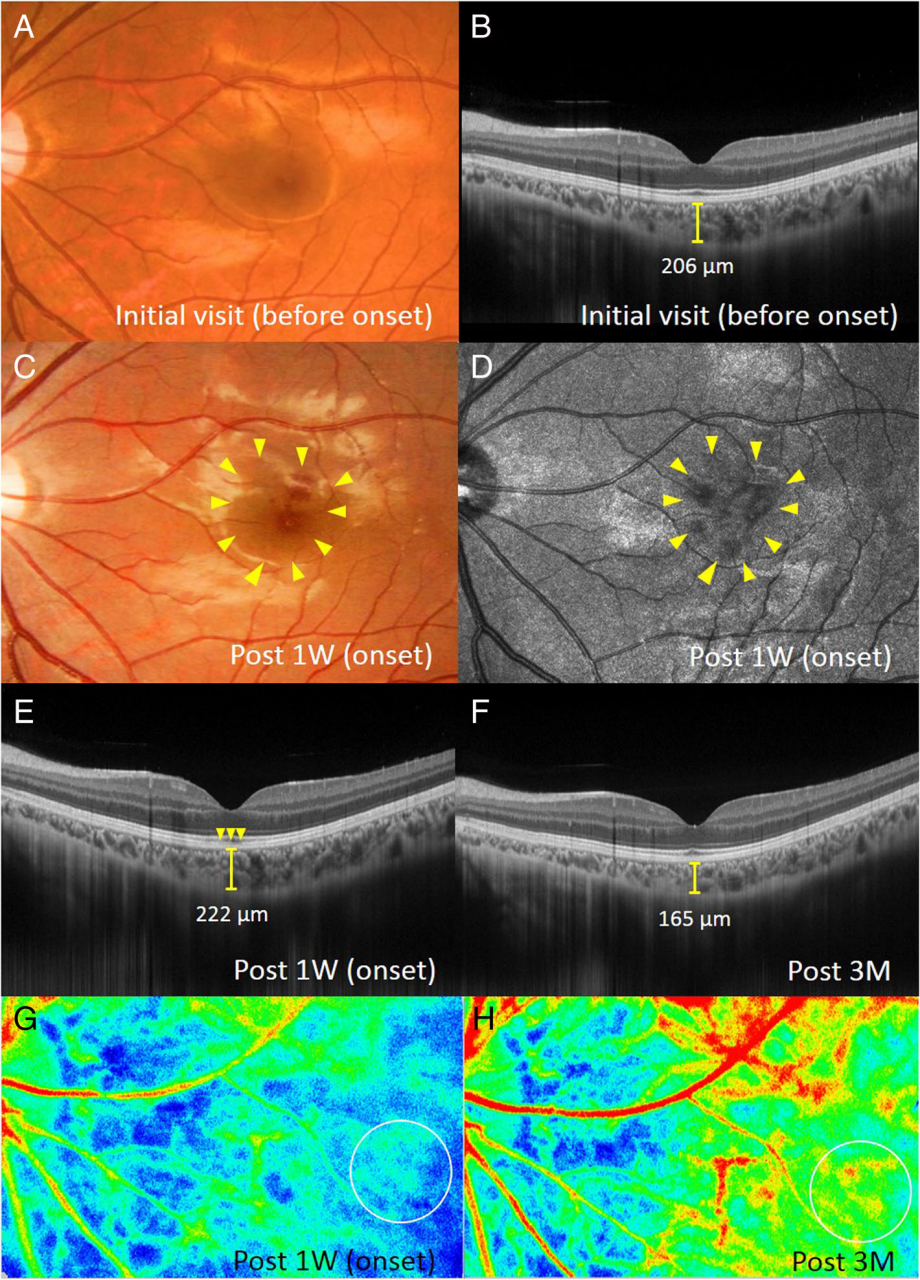
Images of the left eye at the first visit (**a**, **b**), 1 week (**c**-**e**, **g**), and 3 months after (**f**, **h**) the first visit in a patient with bilateral AMN. **a** Fundus photograph shows no abnormal retinal appearance. **b** EDI-OCT shows normal retinal structures at the macula. CCT is 206 μm. **c** Fundus photograph shows the development of a wedge-shaped dark reddish lesion (arrows) at the macula. **d** SLO infrared imaging shows the dark area corresponding to the macular lesion. **e** A horizontal EDI-OCT image through the fovea shows the disruption of IZ at the fovea (arrows). CCT increased to 222 μm, compared with that at the first visit. **f** CCT decreased to 165 μm with recovery of the foveal IZ. **g**, **h** On composite color map images of LSFG, MBR within the circle at 3 months after the first visit (**h**) increased by 68.3% compared with that at the AMN onset (**g**)

The patient’s best-corrected visual acuity was 1.2 OU with no refractive error. Slit-lamp examination revealed no abnormal findings OU. Funduscopic examination revealed a wedge-shaped dark reddish lesion at the macula OD and no abnormal findings OS (Figs. 1a and 2a). Scanning laser ophthalmoscope (SLO) infrared imaging showed a dark area corresponding to the dark reddish lesion (Fig. 1b). EDI-OCT showed loss or disruption of the interdigitation zone (IZ) at the macula OD and normal findings OS (Figs. 1e and 2b). The patient was diagnosed with AMN OD.

On the next day following the first visit, the patient complained of central vision loss OS. One week after the first visit, a wedge-shaped dark reddish lesion was observed at the macula OS (Fig. 2c, d). EDI-OCT showed the disruption of the IZ at the fovea OS (Fig. 2e). Fluorescein angiography revealed hypofluorescence from the initial to late phases corresponding to the lesion OU (Fig. 1c). On indocyanine green angiography, initial geographic hypofluorescence was observed corresponding to the AMN lesion OU (Fig. 1d), but the finding became obscure at the late phase. Fundus autofluorescence (FAF) revealed faint hypo-autofluorescence, corresponding to the lesion OU. Humphrey threshold 10–2 perimetry showed decreased central sensitivity corresponding to the lesion. The patient was diagnosed with AMN OU and was monitored with no treatment. Three months after the first visit, the dark reddish lesions and a dark area on SLO improved OU. EDI-OCT showed recovery of the macular IZ (Figs. 1f and 2f). On FAF, hypo-autofluorescence resolved OU.

Using LSFG-NAVI (Softcare, Fukuoka, Japan) and EDI-OCT (RS-3000 Advance; NIDEK, Gamagori, Japan), central choroidal thickness (CCT) and mean blur rate (MBR) at the macula were sequentially measured.

EDI-OCT measurements were obtained from both eyes at the initial visit, 1 and 3 weeks, and 1 and 3 months after the first visit. CCT was determined by manually measuring the distance between the outer border of the hyper-reflective line corresponding to the retinal pigment epithelium and the outer border of the choroid (Figs. 1e, f and 2b, e, f), using a horizontal scan through the fovea (scan length, 12.0 mm). Two authors (Yuki. H., Yuka. H.), blinded to clinical information, independently evaluated EDI-OCT images and the average values of two authors were compared.

LSFG was conducted 5 consecutive times at 1 and 3 weeks and 1 and 3 months. The circles used in obtaining these images were set to the AMN lesion site (Figs. 1g, h and 2g, h). The positioning of the circles was decided manually by comparing fundus photography with LSFG color map. The average MBR values were calculated for each phase using the LSFG Analyzer software (v 3.0.47; Softcare, Fukuoka, Japan), which allowed automatic setting of each circle during the follow-up visit at the same site where a circle was previously set at baseline to ensure consistency. Sequential changes in average MBR were evaluated as the changing rates of average MBR against the first measurement values, because MBR is a quantitative index of the “relative” blood flow velocity.

Within a certain range, there is a linear relationship between choroidal blood flow and OPP in healthy subjects [6, 22]. To exclude the possibility of such physiological responses from the current results, OPP was calculated from the patient’s blood pressure and intraocular pressure, as described previously [7].

**Fig. 3:**
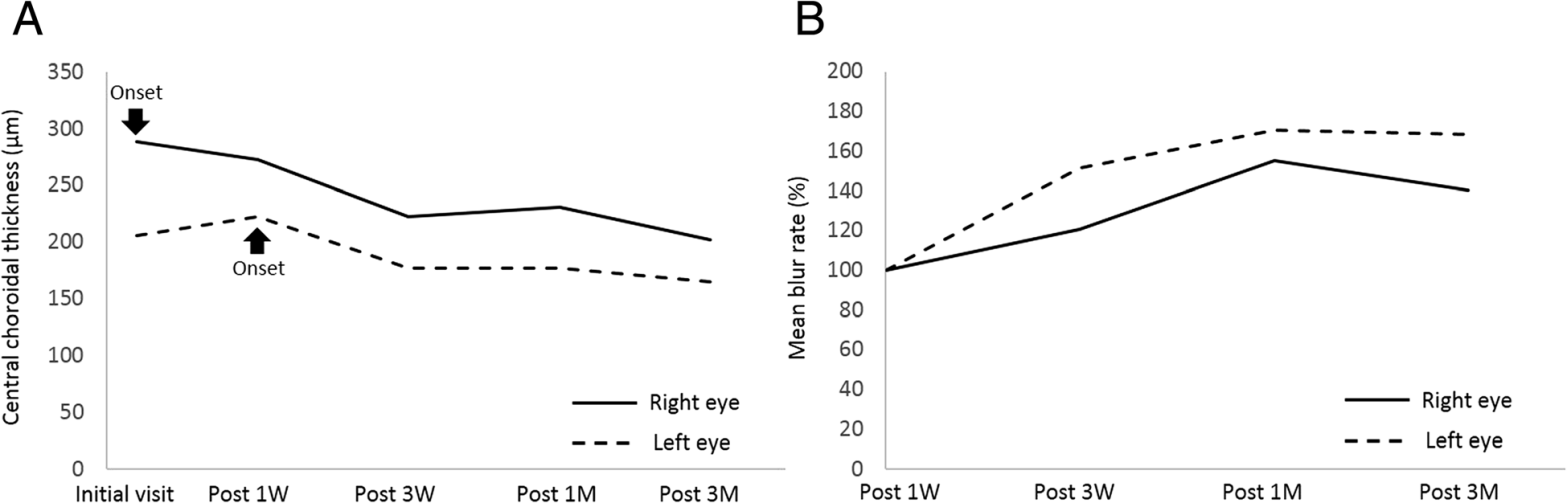
The graph shows changes in the CCT (**a**) and MBR values (**b**) during the 3-month follow-up period. **a** When compared with those at the AMN onset, CCT at 3 months decreased by 86 μm in the right eye and 57 μm in the left eye. **b** Compared with the baseline measurement values (after 1 week), macular MBR increased by 20–55% in the right eye and 51–71% in the left eye

CCT changes in the present case are shown in Fig. 3a. In the right eye, CCT decreased from 288.0 μm at the first visit (at the acute stage of AMN) to 272.0, 222.0, 231.0, and 202.0 μm at 1 week, 3 weeks, 1 month, and 3 months later, respectively (Fig. 1e, f and 3a). In the left eye, CCT increased from 206.0 μm at the first visit (before the onset of AMN) to 222.0 μm after 1 week (at the acute stage of AMN, Fig. 2b, e) and decreased to 177.0, 177.0, and 165.0 μm at 3 weeks and 1 and 3 months after the first visit, respectively (Figs. 2f and 3a).

The LSFG color map obtained 3 months after baseline clearly demonstrated that the MBR values at the lesion sites were higher than baseline data in both eyes (Fig. 3b). The average MBR values were as follows: 6.9 ± 1.2, 8.3 ± 0.8, 10.7 ± 0.6, and 9.7 ± 0.6 OD; 6.0 ± 0.4, 9.1 ± 0.6, 10.3 ± 0.6, and 10.1 ± 1.0 OS at baseline, 3 weeks and 1 and 3 months, respectively. When the rate of change in MBR was evaluated using the MBR values at baseline, 20.4, 54.9, and 40.0% increments were noted OD at 3 weeks, 1 month, and 3 months, respectively. Similarly, 51.1, 70.6, and 68.3% increments were noted OS at 3 weeks, 1 month, and 3 months, respectively. OPP slightly decreased from 31.0 mmHg OD and 34.0 mmHg OS at baseline to 26.4 mmHg OD and 27.4 mmHg OS at 3 months.

## Discussion and conclusions

In a patient with bilateral AMN, we observed the following results: (1) In the right eye, CCT was 82 μm higher at the onset of AMN than the value prior to the onset of AMN OS and decreased by 86 μm with regression of AMN; (2) In the left eye, CCT increased by 16 μm at the onset of AMN compared with the CCT prior to the onset and decreased by 57 μm after 3 months; (3) Macular MBR increased by 20–55% OD and 51–71% OS from baseline to 3 months after the onset of AMN.

We speculate that there are two possible explanations for the mechanism underlying the sequential reduction in choroidal thickness in the present case; one is choroidal thinning (permanent volume loss) during the clinical course, and the other is transient choroidal thickening at the AMN onset. We found that the choroid in the left eye was thicker at the acute stage of AMN than before the onset. Since funduscopy, OCT, and FAF imaging during the AMN regression did not show any findings suggestive of chorioretinal atrophy and there was improvement of visual function and outer retinal morphology, we suggest that the choroid at the lesion site thickened with the development of AMN in this case.

In the present case, MBR reduced at the AMN lesion sites at the acute stage of AMN, as previously reported [14, 15]. These results suggest that a disturbance in choroidal circulation occurs at the choroidal deep layers of the AMN lesion site, as MBR reflects blood flow velocity mainly at the choroidal deep layers [23, 24]. The presence of hypofluorescence observed at the initial phase of indocyanine green angiography may support this observation. OCTA findings in this disease indicated a reduction in the flow of the choriocapillaris [5]. Considering all of our results and the previous observations, we suggest that patients with AMN have blood flow impairments at the level of not only the choriocapillaris but also the choroidal deeper layers. In patients with AMN, therefore, the photoreceptor impairment may be due to choroidal involvement, because the choriocapillaris supplies oxygen and nutrients to the photoreceptors.

In this study, we confirmed that the morphological and circulatory patterns of the choroid in AMN were similar to those of choroiditis-causing conditions such as Vogt-Koyanagi-Harada disease, and may similarly be called an inflammatory pattern [7–17]. Therefore, our data indicated that inflammatory choroidal swelling (i.e.*,* thickning) with circulatory disturbance might be related to the pathogenesis of AMN. In patients with AMN, previous reports showed improvement of visual functions after administration of systemic corticosteroid therapy, which also supports the possible inflammatory etiology of AMN.

In conclusion, EDI-OCT results in a patient with AMN during a 3-month period revealed that the choroid at the macula thickened with the development of AMN. Moreover, CCT decreased and choroidal blood flow velocity increased with regression of AMN. These results further strengthened our hypothesis that the inflammatory circulation disturbance in the thickened choroid plays a role in the pathogenesis of AMN.

## Abbreviations

AMN: Acute macular neuroretinopathy
CCT: Central choroidal thickness
EDI-OCT: Enhanced depth imaging optical coherence tomography
FAF: Fundus autofluorescence
IZ: Interdigitation zone
LSFG: Laser speckle flowgraphy
MBR: Mean blur rate
OCTA: Optical coherence tomography angiography OPP: ocular perfusion pressure
SD-OCT: Spectral-domain optical coherence tomography
SLO: Scanning laser ophthalmoscope

## References

[1] Bos PJ, Deutman AF (1975). Acute macular neuroretinopathy. Am J Ophthalmol.

[2] Bhavsar KV, Lin S, Rahimy E, Joseph A, Freund KB, Sarraf D (2016). Acute macular neuroretinopathy: a comprehensive review of the literature. Surv Ophthalmol.

[3] Neuhann IM, Inhoffen W, Koerner S, Bartz-Schmidt KU, Gelisken F (2010). Visualization and follow-up of acute macular neuroretinopathy with the Spectralis HRA + OCT device. Graefes Arch Clin Exp Ophthalmol.

[4] Yeh S, Hwang TS, Weleber RG, Watzke RC, Francis PJ (2011). Acute macular outer retinopathy (AMOR): a reappraisal of acute macular neuroretinopathy using multimodality diagnostic testing. Arch Ophthalmol.

[5] Lee SY, Cheng JL, Gehrs KM, Folk JC, Sohn EH, Russell SR (2017). Choroidal features of acute macular neuroretinopathy via optical coherence tomography angiography and correlation with serial multimodal imaging. JAMA Ophthalmol.

[6] Akahori T, Iwase T, Yamamoto K, Ra E, Terasaki H (2017). Changes in choroidal blood flow and morphology in response to increase in intraocular pressure. Invest Ophthalmol Vis Sci.

[7] Hirooka K, Saito W, Namba K, Takemoto Y, Mizuuchi K, Uno T, Tagawa Y (2015). Relationship between choroidal blood flow velocity and choroidal thickness during systemic corticosteroid therapy for Vogt-Koyanagi-Harada disease. Graefe's Arch Clin Exp Ophthalmol.

[8] Takahashi A, Saito W, Hashimoto Y, Saito M, Ishida S (2014). Impaired circulation in the thickened choroid of a patient with serpiginous choroiditis. Ocul Immunol Inflamm.

[9] Saito M, Saito W, Hashimoto Y, Yoshizawa C, Shinmei Y, Noda K (2014). Correlation between decreased choroidal blood flow velocity and the pathogenesis of acute zonal occult outer retinopathy. Clin Exp Ophthalmol.

[10] Hashimoto Y, Saito W, Saito M, Hasegawa Y, Takita A, Mori S (2017). Relationship between choroidal thickness and visual field impairment in acute zonal occult outer retinopathy. J Ophthalmol.

[11] Hashimoto Y, Saito W, Saito M, Hirooka K, Mori S, Noda K (2015). Decreased choroidal blood flow velocity in the pathogenesis of multiple evanescent white dot syndrome. Graefe's Arch Clin Exp Ophthalmol..

[12] Hashimoto Y, Saito W, Saito M, Hasegawa Y, Mori S, Noda K (2016). Relationship between choroidal thickness and visual impairment in multiple evanescent white dotsyndrome. Acta Ophthalmol.

[13] Hirooka K, Saito W, Hashimoto Y, Saito M, Ishida S (2014). Increased macular choroidal blood flow velocity and decreased choroidal thickness with regression of punctate inner choroidopathy. BMC Ophthalmol.

[14] Hashimoto Y, Saito W, Mori S, Saito M, Ishida S (2012). Increased macular choroidal blood flow velocity during systemic corticosteroid therapy in a patient with acute macular neuroretinopathy. Clin Ophthalmol.

[15] Hirooka K, Saito W, Noda K, Ishida S (2014). Enhanced-depth imaging optical coherence tomography and laser speckle flowgraphy in a patient with acute macular neuroretinopathy. Ocul Immunol Inflamm.

[16] Hashimoto Y, Saito W, Saito M, Hirooka K, Mori S, Noda K (2015). Increased choroidal blood flow velocity with regression of unilateral acute idiopathic maculopathy. Jpn J Ophthalmol.

[17] Hirooka K, Saito W, Saito M, Hashimoto Y, Mori S, Noda K (2016). Increased choroidal blood flow velocity with regression of acute posterior multifocal placoid pigment epitheliopathy. Jpn J Ophthalmol.

[18] Saito M, Saito W, Hashimoto Y (2013). Macular choroidal blood flow velocity decreases with regression of acute central serous chorioretinopathy. Br J Ophthalmol.

[19] Saito M, Noda K, Saito W, Ishida S (2018). Relationship between choroidal blood flow velocity and choroidal thickness in patients with regression of acute central serous chorioretinopathy. Graefes Arch Clin Exp Ophthalmol.

[20] Hashimoto R, Hirota A, Maeno T (2016). Choroidal blood flow impairment demonstrated using laser speckle flowgraphy in a case of commotio retinae. Am J Ophthalmol Case Rep.

[21] Ishikawa Y, Hashimoto Y, Saito W, Ando R, Ishida S (2017). Blood flow velocity and thickness of the choroid in a patient with chorioretinopathy associated with ocular blunt trauma. BMC Ophthalmol.

[22] Riva CE, Titze P, Hero M, Petrig BL (1997). Effect of acute decreases of perfusion pressure on choroidal blood flow in humans. Invest Ophthalmol Vis Sci.

[23] Isono H, Kishi S, Kimura Y, Hagiwara N, Konishi N, Fujii H (2003). Observation of choroidal circulation using index of erythrocytic velocity. Arch Ophthalmol.

[24] Watanabe G, Fujii H, Kishi S (2008). Imaging of choroidal hemodynamics in eyes with polypoidal choroidal vasculopathy using laser speckle phenomenon. Jpn J Ophthalmol.

